# Metabolic Differences in Neuroimaging with [^18^F]FDG in Rats Under Isoflurane and Hypnorm–Dormicum

**DOI:** 10.3390/tomography11010004

**Published:** 2025-01-03

**Authors:** Aage Kristian Olsen Alstrup, Mette Simonsen, Kim Vang Hansen, Caroline C. Real

**Affiliations:** Department of Nuclear Medicine & PET, Aarhus University Hospital, 8200 Aarhus, Denmark; metsimon@rm.dk (M.S.); .

**Keywords:** anesthesia, [^18^F]FDG, imaging, neuroscience, rats, PET/MR

## Abstract

Background: Anesthesia can significantly impact positron emission tomography (PET) neuroimaging in preclinical studies. Therefore, understanding these effects is crucial for accurate interpretation of the results. In this experiment, we investigate the effect of [^18^F]-labeled glucose analog fluorodeoxyglucose ([^18^F]FDG) uptake in the brains of rats anesthetized with two commonly used anesthetics for rodents: isoflurane, an inhalation anesthetic, and Hypnorm–Dormicum, a combination injection anesthetic. Materials and Methods: Female adult Sprague Dawley rats were randomly assigned to one of two anesthesia groups: isoflurane or Hypnorm–Dormicum. The rats were submitted to dynamic [^18^F]FDG PET scan. The whole brain [^18^F]FDG standard uptake value (SUV) and the brain voxel-based analysis were performed. Results: The dynamic [^18^F]FDG data revealed that the brain SUV was 38% lower in the isoflurane group after 40 min of image (2.085 ± 0.3563 vs. 3.369 ± 0.5577, *p* = 0.0008). In voxel-based analysis between groups, the maps collaborate with SUV data, revealing a reduction in [^18^F]FDG uptake in the isoflurane group, primarily in the cortical regions, with additional small increases observed in the midbrain and cerebellum. Discussion and Conclusions: The observed differences in [^18^F]FDG uptake in the brain may be attributed to variations in metabolic activity. These results underscore the necessity for careful consideration of anesthetic choice and its impact on neuroimaging outcomes in future research.

## 1. Introduction

Positron emission tomography (PET) scanning of rats and other laboratory animals is traditionally carried out under full anesthesia, primarily to ensure that the animals lie still during the entire scan but also to ensure good animal welfare [1]. However, anesthesia may have major effects on PET neuroimaging in animal experimentation, as reviewed by Alstrup and Smith (2013) [2]. This is not surprising since it is obviously impossible to anesthetize the brain without affecting its physiology. Furthermore, PET imaging often takes up to several hours, and thus, there is plenty of time for major physiological effects to happen that may affect the PET scans. The length of anesthesia covers not only the PET scan time but also the time for preparation, including surgical placement of catheters, MR pre-scan, and waiting time before the production and injection of radiotracers. Alternatives for avoiding anesthesia have been tested, including building a special small PET scanner (the RatCAP) that freely moving rats can wear on their heads [3]. Also, motion tracking of awake rats has been tested [4,5,6], and others have tried to fix the rat’s head to the scanner during imaging [7]. It has been shown that the fixation of rat heads leads to stress [8], which has a major impact on PET imaging studies [9]. Since the stress level is different from one fixated rat to another, this could add to variation and require that the group sizes be increased [9]. Fixation during PET imaging thus goes against both refinement and reduction in the 3R concept for the ethical use of laboratory animals in research [10]. Even for preclinical PET studies where the fixated animals have been trained in head-restraining devices prior to scanning, it is rarely documented that they, in fact, are not stressed. These findings suggest that while awake imaging introduces technical challenges, it is valuable for studies requiring unaltered physiological conditions. Anesthesia remains practical for studies where immobilization is critical, but researchers must carefully choose protocols to minimize confounding effects.

An obvious approach to this problem is to accept that the animals must be anesthetized and that the researchers, therefore, must be aware of the anesthetic side effects on the imaging results. With such an approach, one must study and know these effects of anesthesia and discuss them in the published papers as potential limitations. Today, most preclinical PET studies are, in fact, performed on anesthetized experimental animals. Several research groups have actively investigated anesthetic effects in experimental animals used for PET neuroimaging ([11]; reviewed in [2]). Such studies have shown that anesthesia may very often affect the uptake and distribution of the neuro-tracers in several different ways. Anesthetics may affect certain specific molecular mechanisms in the brain that can change the uptake of specific tracers [12]. However, it may be more common that the anesthetics affect the cerebral blood flow (CBF) and brain metabolism of the animals, which indirectly also affects tracer kinetics [2]. Anesthesia is well known to have major effects on cerebral blood flow, as it can be twice as high under isoflurane than under propofol anesthesia of rats [13]. Higher cerebral blood flow means a greater supply of the tracer to the brain, and thus, an increased initial uptake must be expected. Especially in the case of irreversible binding tracers, this can be crucial since the tracer remains bound in the brain—as opposed to reversibly binding tracers, where part of the tracer will leave the brain again. The cerebral blood flow is thus strongly influenced by the anesthetic, which correspondingly also affects the brain’s metabolism. In the case of the [^18^F]-marked glucose analog, fluorodeoxyglucose [^18^F]FDG, the neurons’ uptake of the tracer is directly related to the metabolism in the brain [14]. Most anesthetics lower the brain metabolism, as shown by 22–65 percent reduced cerebral [^18^F]FDG uptake [15,16,17], but also unchanged [^18^F]FDG uptake has been observed in dogs during medetomidine–tiletamine–zolazepam anesthesia compared to awake state [18]. Another study performed in Göttingen minipigs revealed that the standard uptake value (SUV) of [^18^F]FDG was higher during propofol than during ketamine–tiletamine–zolazepam mixture or propofol/isoflurane combined anesthesia [19]. The changed [^18^F]FDG uptake is not just due to the effects of depression from anesthesia on brain metabolism but may also be due to the indirect effects of changed blood glucose and blood lipids in the animals. A study that evaluated the morphine self-administered effects in rat brains with [^18^F]FDG observed totally different results when the animals were kept awake or under isoflurane after [^18^F]FDG administration. Isoflurane anesthesia, as compared to the awake condition, reduced brain glucose metabolism in the olfactory, cortex, thalamus, and basal ganglia while increasing brain glucose metabolism in the midbrain, hypothalamus, hippocampus, and cerebellum [20]. There are, therefore, a number of different mechanisms by which anesthesia can affect the trace substances in the brain, and it is therefore necessary in each case to investigate the combination of anesthesia and trace substances in order to obtain certain knowledge.

In principle, anesthesia effects can be investigated in three different ways, all of which have advantages and disadvantages [2]. The most common approach is to compare radiotracer uptake and distribution in animals anesthetized with different anesthetics. The animals are scanned with the same radiotracer but anesthetized with different drugs or drug combinations. As an example, we earlier compared the striatal uptake of the dopamine receptor D_2_/D_3_ antagonist [^11^C]raclopride in rats anesthetized with isoflurane or fentanyl–fluanisone–midazolam, known as Hypnorm–Dormicum [21]. The two forms of anesthesia were chosen as they are particularly well used for anesthetizing mice and rats. The striatal binding potential of [^11^C]raclopride was more than twice as high during isoflurane compared to Hypnorm–Dormicum anesthesia, but without the possibility of clarifying which anesthesia was closest to the awake stage, which is obviously the disadvantage of this comparing anesthesia approach. However, our study documented that the anesthetic effect can be greater than typical interventions in animal studies, such as treatment with d-amphetamine [22,23]. The advantage of the comparing anesthesia approach is that studies are relatively easy to perform and allow dynamic data. Furthermore, it can be performed as a non-recovery study ensuring good animal welfare. The second approach compares awake and anesthetized animals scanned with the same tracer, but it typically requires that the awake animals are fixated during the imaging and are, therefore, more likely to reflect stressed awake animals [15]. Although this approach appears to provide data for the awake state, the results can be both ethically and scientifically objectionable. A third approach involves scanning both anesthetized and freely moving awake animals with the same tracer. Instead of fixating the animals, they are anesthetized at different time points after tracer injection, and immediately afterward, they are PET scanned [24]. For example, in this study, the effects of acute cocaine exposure on [^18^F]FDG uptake were observable only when animals were maintained under isoflurane anesthesia. Cocaine increases locomotor activity, so when animals were injected and allowed free movement in the cage, heightened metabolism likely shifted toward muscle tissue [25]. This makes real dynamic data for each animal impossible, but instead, data are obtained at different times performed on groups of animals. A disadvantage is therefore obvious: the high number of animals needed in such studies.

Numerous combinations of anesthetics and PET tracers warrant investigation. In this study, we focused on two well-established anesthetic methods: isoflurane, administered via inhalation, and the rodent anesthetic mixture Hypnorm–Dormicum, which is commonly used in European laboratories for rat anesthesia [21]. However, we are not aware that any comparisons between isoflurane and Hypnorm–Dormicum anesthesia should have been made except in our own study [21]. This is consistent with a PubMed search (performed on 11 November 2024), which did not yield such results. We specifically examined the effects of these anesthetics on [^18^F]FDG uptake, as it is likely the most widely utilized PET tracer worldwide. [^18^F]FDG is extensively employed in preclinical studies assessing metabolism, cancer, and infections [26,27], making it an important target for evaluating the impact of anesthesia on neuroimaging outcomes. The rat was chosen as it is one of the two most used experimental animals in the world and is very often used as an experimental animal in PET studies. A Pubmed search on Pubmed (conducted online on 6 November 2024) on the keywords [^18^F]FDG and *rats* thus yields 1290 search results, and of these, a total of 713 were related to the keyword *brain*. The study seeks to clarify how different anesthetic protocols affect brain metabolism in preclinical models using [^18^F]FDG.

## 2. Materials and Methods

### 2.1. License, Power and Design

The study was performed in accordance with 2010/63/EU and the Danish Animal Experimental Act on a license granted by The Danish Experimental Animal Inspectorate (license number 2022-15-0201-01182). To calculate the needed number of rats, we chose six rats in each group based on the resource equation: E = [number of animals] − [number of groups] = 12 − 2 = 10, where *E* should be between 10 and 20 animals, to avoid overuse of animals, but ensure statistical power [28]. This approach can be used as an approximation when variation (like in our study) is not known. In addition, N = 6 is a typical group size in many preclinical publications and therefore also relevant here. The rats were randomly anesthetized with either isoflurane (N = 6) or Hypnorm–Dormicum (N = 6) and dynamic PET scanned for one hour after intravenous [^18^F]FDG injections. The [^18^F]FDG uptake in the brains was compared between the two groups.

### 2.2. Animals and Housing

Twelve barrier-raised female Sprague Dawley rats (Janvier-Labs, Le Genest-Saint-Isle, France) weighing 250–306 g were acclimatized for at least one week prior to the study. The rats had undergone health monitoring according to the Federation of European Laboratory Animal Science guidelines (FELASA) [29]. The animals were housed in groups of 2–3 in IVC-cages (Techniplast, Buguggiate, Italy) on aspen bedding (Finn Tapvei, Kaavi, Finland) at 20–24 °C and at a relative humidity of 45–65% and a light–dark cycle of 12 h (light on from 6 AM to 6 PM). The animals had free access to tap water and a standard laboratory pellet diet (Altromin 1324, Aarhus, Denmark) until start of anesthesia. The rats were weighed on the day of the experiment. No fasting was performed.

### 2.3. Anesthesia and Monitoring

The twelve rats were weighed individually and either anesthetized by inhalation (N = 6) or injection (N = 6). The anesthesia was performed according to the procedures commonly used at Aarhus University and several other similar research sites: Inhalation anesthesia was induced in an induction chamber with 5% isoflurane (Forene, Abbott, Solna, Sweden) in oxygen until deep sedation after 1–3 min, and then on nose masks (2% for maintaining) in a gas mixture (0.4-L O_2_ and 1.5-L medical air) with spontaneous respiration. Injection anesthesia was induced with subcutaneous injection (sc) of Hypnorm–Dormicum mixture (Hypnorm, VetaPharma, Leeds, United Kingdom; Midazolam, Hamelm, Herlev, Denmark). A mixture contained 0.02 mg fentanyl, 0.75 mg fluanisone, and 0.38 mg midazolam SC, and the induction dose was 0.25 mL supplemented with 0.1 mL approximately every 20–30 min when the tail reflex was positive. The same gas mixture (0.4-L O_2_ and 1.5-L medical air) was used for these rats, and they were also on spontaneous respiration. A 24 G 0.7–19 mm BD Neoflon (BD, Eysins, Switzerland) was placed in the tail vein for tracer injection. Pulse, respiration frequency, and body temperature were monitored (Medico MultiCell, Imaging Chamber) during the PET imaging.

### 2.4. [^18^F]FDG PET/MR Imaging

The rat was placed in the Mediso nanoScan^®^ PET/MRI 1 T scanner (Mediso Ltd., Budapest, Hungary), briefly MRI-scanned (for attenuation correction), and then PET scanned for 60 min after administration of [^18^F]FDG (18.7–35.2 MBq in 0.2–0.3 mL) intravenously in the tail catheter (the catheter was flushed with 0.1–0.2 mL saline after tracer injection). The rats were euthanized at the end of the study by an overdose (100 mg/kg) of pentobarbitone in the vein catheter.

### 2.5. PET Data Analyses

Emission sinograms were iteratively reconstructed into a 23-frames (8 × 15, 4 × 30, 2 × 60, 2 × 120, 4 × 300, 3 × 600 s) and one frame of 20 min in the last part of the acquisition (Tera Tomo 3D, 4 iteration and 6 subsets, as reconstruction method) after being submitted to MRI attenuation correction, normalized, and corrected for scatter and radioactivity decay. PET image analysis was performed with PMOD software (PMOD™ Technologies Ltd., Zurich, Switzerland, version 4.0). [^18^F]FDG PET images were registered to MRI template, and whole brain volume of interest (VOI), available on PMOD software, was applied for brain analysis.

The brain radioactivity concentration was calculated in the whole brain and expressed as standardized uptake value (SUV): [tissue activity concentration (kBq/mL) × body weight (g)]/[injected dose (kBq)] for each region. A tissue density of 1 g/mL was assumed.

In addition, the SUV average images were normalized for whole brain uptake to generate SUVR images. We also generated SUV % of different images to highlight the regions with most difference between the groups ((Hypnorm–Dormicum − Isoflurane)/Isoflurane × 100).

To assess the effects of anesthesia on other organs, which is not the focus of neuroimage, we also evaluated the heart uptake, which was included in the same field of view (FOV) as the brain. For this purpose, we performed the analysis using the PMOD^TM^ View tool, employing the automated hot 3D VOI tool to delineate the VOI for each animal’s heart. The data for the SUV curve and the average SUV for the 40–60 min interval, handled as static images, were included in the analysis.

### 2.6. Voxel-Based Analysis

Voxel-based analyses were performed in Statistical Parametric Mapping, version 12 (SPM12; https://www.fil.ion.ucl.ac.uk/spm/, accessed/installed on 27 September 2023), using SUV [^18^F]FDG images in combination with the Small Animal Molecular Imaging Toolbox (SAMIT; http://mic-umcg.github.io/samit/, accessed/installed on 2 September 2024) package. All the images were spatially normalized using affine transformations to T2 MRI template rat brain in Paxinos space and smoothed with a 0.8 mm isotropic Gaussian kernel. To ensure that the analysis contained only voxels mapping the rat brain, the differences between the global uptake of images were adjusted using the proportional scaling option of SPM12. For the interpretation of group differences, T map thresholds were set at ≤0.001, uncorrected for multiple comparisons, and the extent threshold ≥ 200 voxels for the cluster size (kE). Only clusters with *p* ≤ 0.05, corrected for family-wise error (FWE), were considered significant [30].

### 2.7. Statistics

The data were submitted to Shapiro–Wilk test normality test. The data that were normally distributed were submitted to parametric tests. Student’s *t*-test was used to compare two groups, and comparisons between the curve were submitted to two-way analysis of variance (ANOVA), and anesthesia and time after injection were the variables. ANOVA was followed by Šidák correction when appropriate. Each *p* value was adjusted to account for multiple comparisons. A *p*-value of <0.05 was considered significant.

## 3. Results

### 3.1. General Study Information

There were no statistical differences in the rats’ weights and [^18^F]FDG doses in the two groups, and there were also no statistical differences in the monitoring parameters pulse, respiration rate, and body temperature (all *p*-values were between 0.113 and 0.937 (see Table 1) between the two groups.

### 3.2. [^18^F]FDG PET Imaging—Standardized Uptake Value (SUV)

#### 3.2.1. Brain Uptake

**Figure 1:**
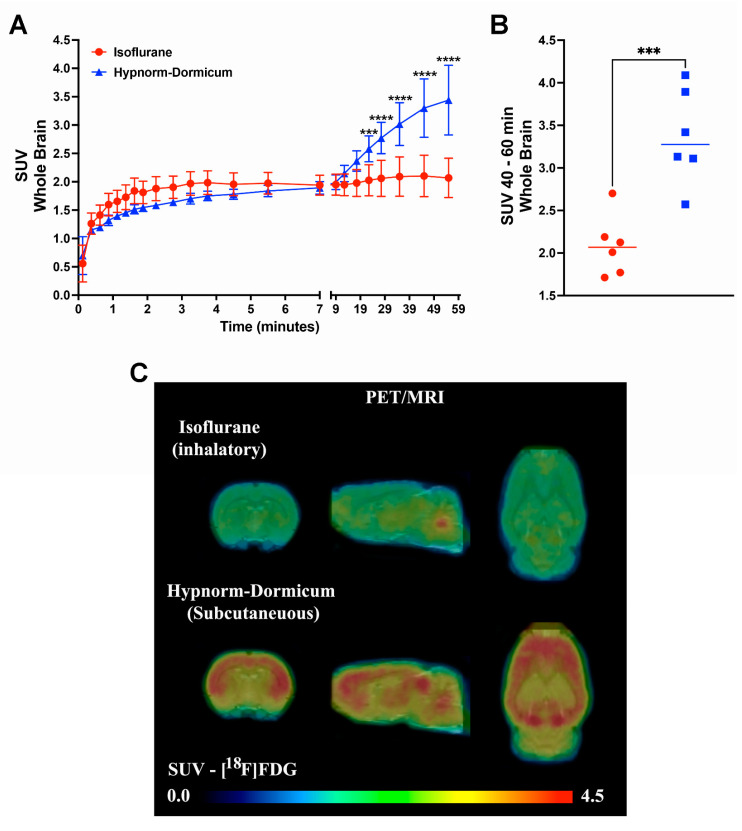
Dynamic PET data from rats scanned with [^18^F]FDG under either isoflurane (N = 6) or Hypnorm–Dormicum (N = 6) anesthesia. The whole brain standard uptake value (SUV) is shown as a function of time after tracer injection (**A**). One frame PET data (20 min of image acquisition between 40 and 60 min after tracer injection) is shown as a graph (**B**) and as a mean SUV PET imaging (**C**). ***: *p* < 0.001; ****: *p* < 0.0001.

**Figure 2:**
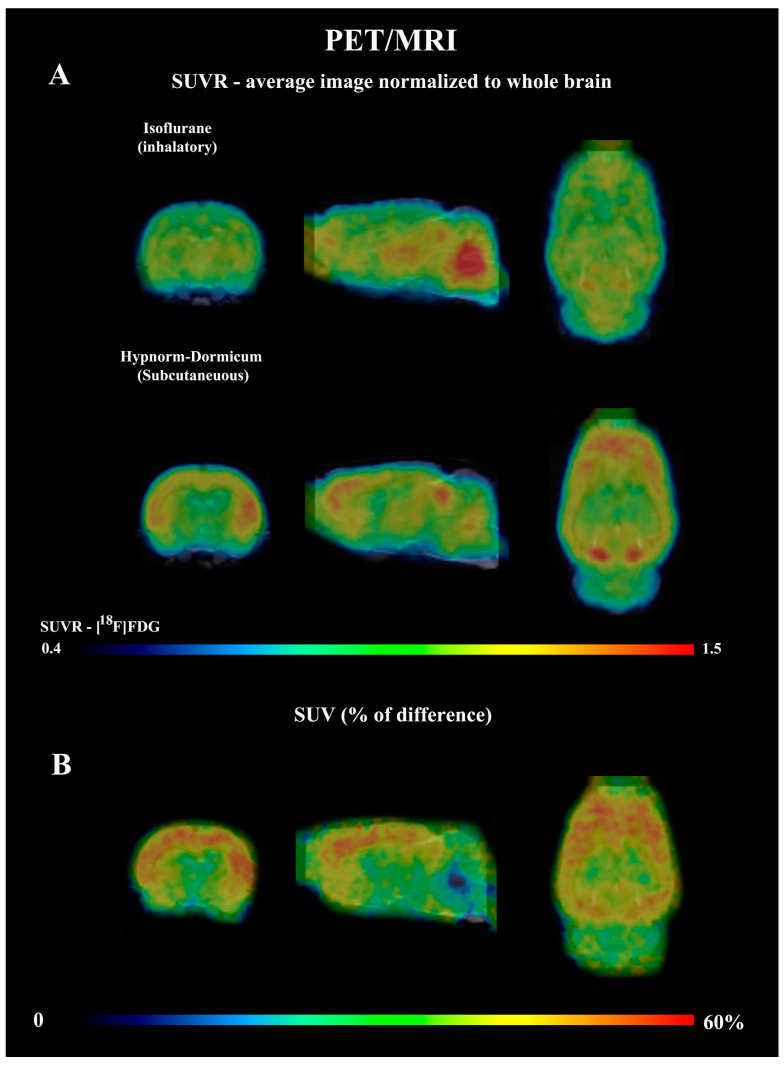
Average PET data normalized to whole brain standard uptake value (SUVR) from rats scanned with [^18^F]FDG under either isoflurane (N = 6) or Hypnorm–Dormicum (N = 6). The whole brain (SUVR) images for both groups are shown in (**A**), and the % of difference of SUV between groups is shown in (Hypnorm-Isoflurane)/Isoflurane × 100) (**B**). Note that the [^18^F]FDG uptake is higher in the cortex of Hypnorm–Dormicum and in midbrain and cerebellum of the isoflurane group. The images were generated automatically in the PMOD^TM^ tool. For the whole brain normalized image, the mean value of SUV from all six animals per group was used.

The dynamic PET data revealed that after 25 min, the SUV for isoflurane group was lower than for Hypnorm–Dormicum data (frame 19: −21%, 2.03 ± 0.27 vs. 2.58 ± 0.23, *p* = 0.0009; frame 20: −26%, 2.06 ± 0.32 vs. 2.77 ± 0.23; frame 21: −31%, 2.09 ± 0.35 vs. 3.02 ± 0.38; frame 22: −36%, 2.10 ± 0.39 vs. 3.30 ± 0.51; frame 23: −40%, 2.07 ± 0.35 vs. 3.44 ± 0.61, *p* < 0.0001) (Figure 1A). One frame PET data (20 min of image acquisition between 40 and 60 min after [^18^F]FDG injection) shows an SUV 38% lower in the animals anesthetized with isoflurane after 40 min of acquisition (2.085 ± 0.3563 vs. 3.369 ± 0.5577, *p* = 0.0008) (Figure 1B,C). Figure 2A shows SUVR images, representing the SUV average images normalized to whole-brain uptake, while Figure 2B displays the percentage differences between the Hypnorm–Dormicum and isoflurane groups. The isoflurane group exhibits a marked decrease in [^18^F]FDG uptake in the cortical region compared to the Hypnorm–Dormicum group.

#### 3.2.2. Heart Uptake

**Figure 3:**
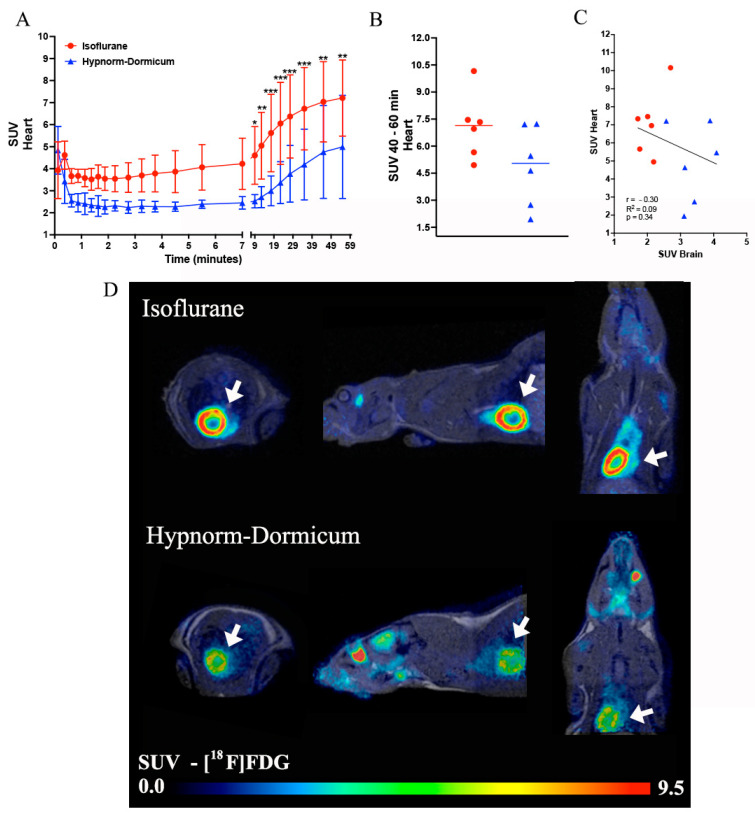
Data representation for heart [^18^F]FDG analysis. The heart SUV curve for isoflurane (in red) and Hypnorm–Dormicum (in blue) groups (**A**). SUV data for the static image from 40 to 60 min after [^18^F]FDG administration (**B**). Data correlation for heart and brain SUV (**C**). Illustrative image of PET/MRI [^18^F]FDG uptake in SUV, the white arrows indicate the heart uptake. Note that there is a higher uptake in isoflurane animals when compared to Hypnorm–Dormicum animal (**D**). * *p* < 0.05; ** *p* < 0.01; *** *p* < 0.001.

For the heart analysis, as an extra organ analyzed here, the dynamic PET data revealed a higher uptake of [^18^F]FDG in isoflurane animals after 9 min of image (Frame 16—4.60 ± 1.31 vs. 2.52 ± 0.30; Frame 17—5.05 ± 1.52 vs. 2.71 ± 0.48; Frame 18—5.62 ± 1.76 vs. 2.99 ± 0.68; Frame 19—6.05 ± 1.87 vs. 3.37 ± 0.95; Frame 20—6.37 ± 1.89 vs. 3.77 ± 1.28; Frame 21—6.72 ± 1.87 vs. 4.20 ± 1.60; Frame 22—7.04 ± 1.82 vs. 4.76 ± 2.10; Frame 23—7.21 ± 1.73 vs. 5.00 ± 2.34) (Figure 3A). For the static image analysis of the last minutes of the image, we observed a 45% high SUV in the isoflurane group when compared to Hypnorm–Dormicum animals (7.09 ± 1.81 vs. 4.87 ± 2.22; *p* = 0.0869) (Figure 3B,D). The correlation between the brain and heart SUVs was not significant, but we can observe in the graph an inverse profile between the uptake in the heart and in the brain, where we can observe a higher heart uptake and lower brain uptake in isoflurane animals and the opposite effect in the Hypnorm–Dormicum animals (Figure 3C).

### 3.3. [^18^F]FDG PET Imaging—Voxel-Based Analysis—Brain

**Figure 4:**
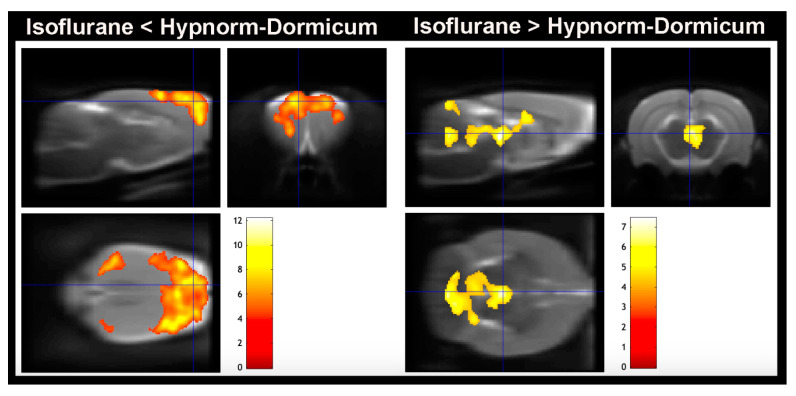
Voxel-based analysis. Lower [^18^F]FDG uptake was observed in the animals anesthetized with isoflurane in somatosensorial cortex, primary motor cortex, and visual cortex when compared to Hypnorm–Dormicum (isoflurane < Hypnorm–Dormicum). On the other hand, there is an increase in [^18^F]FDG uptake in the animals anesthetized with isoflurane in Dorsomedial periaqueductal gray and molecular layer of cerebellum when compared to Hypnorm–Dormicum (isoflurane > Hypnorm–Dormicum. Significance is shown with a T statistic color scale, which corresponds to the level of significance at the voxel level. The data were derived from 6 rats/group.

The voxel-based analysis corroborates the whole brain analysis for SUV, SUVR, and SUV % of difference, and the maps revealed that the high decrease in [^18^F]FDG uptake in the isoflurane animals occurs in the cortex (somatosensorial cortex, primary motor cortex, and visual cortex), and there are some increases in regions of dorsomedial periaqueductal gray and molecular layer of cerebellum when compared to Hypnorm–Dormicum animals (Figure 4 and Table 2).

## 4. Discussion

### 4.1. Overview

Similar to previous studies involving other types of anesthetics and tracers [2], this study demonstrates that anesthetic effects are of importance for preclinical trials involving PET neuroimaging. We found that during the last 25 min of the 60 min scan, AUC was as high as 23% higher for rats anesthetized with Hypnorm–Dormicum compared to isoflurane. Furthermore, a single frame after 45 min showed a 61% higher SUV for this group. These differences are so large that they are at least on par with typical differences in intervention studies. This emphasizes the enormous importance of always anesthetizing experimental animals in a consistent manner.

### 4.2. Possible Causes of the Anesthetic Effects

The obtained differences in [^18^F]FDG uptake in the brain can be due to differences in the brain’s metabolism and in the blood flow, which are influenced by the anesthesia. Cerebral glucose uptake is a measure of the brain’s metabolism, and differences in the uptake of [^18^F]FDG may, therefore, be due to differences in the depressive effect of the two anesthetics on cerebral metabolism. It is well known that isoflurane has a depressant effect on brain metabolism [31]. Hypnorm–Dormicum does not cause the same type of anesthesia as isoflurane, instead, these drugs place the rat in a state of analgesic immobilization, also known as neuroleptanalgesia [32]. This is mainly due to Hypnorm’s content of fluanisone, which is a dissociative drug [32]. For other similar dissociative drugs, such as ketamine, it is well known that brain metabolism is not reduced to the same extent as with traditional anesthesia but can even be increased compared to awake state [33]. The higher cerebral [^18^F]FDG uptake can, therefore, be explained as a higher cerebral metabolism during Hypnorm–Dormicum than under isoflurane anesthesia.

Alternatively, the difference in [^18^F]FDG uptake may theoretically also be due to cerebral blood flow. The question of whether the anesthetic effect is due to differences in CBF between the two groups of rats can be split into two sub-questions. Firstly, whether the two anesthetics cause different levels of CBF, and secondly, whether a difference in CBF can explain differences in [^18^F]FDG uptake. Isoflurane is well-known to increase CBF, which is well-documented in both humans and animals [34,35]. On the other hand, as far as the authors are aware, there is a lack of knowledge about the effect of Hypnorm–Dormicum mixture on CBF in animals. However, it is likely that CBF is considerably lower under Hypnorm–Dormicum anesthesia, as we already suggested when we compared the [^11^C]raclopride uptake in rats under isoflurane and Hypnorm–Dormicum anesthesia [21]. Midazolam (the active drug in Dormicum) lowers CBF in human volunteers [36]. Hypnorm’s effect on CBF is more uncertain, but Eftekhari and coworkers (2020) [37] mention that Hypnorm, like midazolam, reduces CBF. However, the claim was neither documented with data nor references. However, it is reasonable to assume that CBF is higher under isoflurane than under Hypnorm–Dormicum anesthesia. On the other hand, the answer to the second question is probably negative. If CBF has influenced [^18^F]FDG uptake, we would expect that the higher CBF during isoflurane anesthesia would result in the highest brain uptake of the tracer, while the opposite was the case in the cortical areas. It is, therefore, not obvious that the obtained differences in [^18^F]FDG uptake between isoflurane and Hypnorm–Dormicum are mediated through differences in CBF. Also, possible differences in plasma glucose due to isoflurane could have affected the [^18^F]FDG uptake, as reviewed by Mannheim et al. (2017) [38]. In the same article, the authors argue the use of sevoflurane instead of isoflurane, which does not have the same effect on plasma glucose. In addition, a standardized fasting protocol is recommended to use. In the present study, however, the rats were not fasted, but they all had at least a four-hour light period, and it is well known that rats only consume limited food during light periods, as they are nocturnal animals [39]. Whether the rats had a uniform plasma glucose concentration was not investigated in the present study, which required a measurement before and after scanning.

### 4.3. Brain Regions Uptake Difference

The markedly decreased uptake of [^18^F]FDG under isoflurane anesthesia was only valid for the cortical areas (somatosensorial cortex, primary motor cortex, and visual cortex), while the opposite was somewhat the case for regions of midbrain regions and cerebellum. These findings align with previous studies comparing isoflurane with other anesthetics or varying conditions for isoflurane administration [11,20]. In a recent study examining morphine effects, isoflurane anesthesia was associated with reduced brain metabolism in regions such as the olfactory bulb, cortex, basal ganglia, corpus callosum, and thalamus, while metabolism was elevated in the hypothalamus, hippocampus, white matter, midbrain, and cerebellum compared to awake animals following [^18^F]FDG administration [20]. In addition, some studies evaluated the neurons after isoflurane exposure, and after just 15 min of isoflurane, the synaptic and cytoskeletal phosphoproteomes are modulated in the cortex [40]. Together with isoflurane, this induces a functional decrease in excitatory synaptic transmission in prefrontal cortex neurons [41], which can explain the decreased uptake of [^18^F]FDG in cortex regions observed in this study. Additionally, electroencephalography (EEG) studies have shown that isoflurane suppresses the cerebral metabolic rate of oxygen, accompanied by a reduction in high-frequency EEG activity in the cortical area [42]. Magnetic resonance spectroscopy further indicates a 51% decrease in glucose levels in cortical regions [43], which can explain the decreased uptake of [^18^F]FDG in our isoflurane group. Here, we only did an image analysis for [^18^F]FDG, and more studies are necessary to understand the difference between cortical cell activation in Hypnorm–Dormicum versus isoflurane. The increase in isoflurane in the cerebellum of animals can be explained by the olfactory pathways; it is likely that different parts of the cerebellum are active depending on the states such as sensory stimulation or anesthesia, and can increase the [^18^F]FDG, as demonstrated before [44].

### 4.4. Other Parameters

As shown in Table 1, no differences were observed in pulse, respiration rate, and temperature between the two groups of rats. Furthermore, the rats were all kept negative for an interdigital test, and they all made it through to the end of the scan. It can often be difficult to ensure a uniform level of anesthesia, especially when comparing inhalation and injection anesthesia—while the first is inhaled continuously, the second is given as single injections, whereby differences in anesthetic depths can, in principle, arise. Immediately, the identical monitoring parameters (pulse, respiration rate, and temperature) indicate that the two groups of rats were anesthetized at the same level. However, there may still be underlying differences in anesthesia levels. Figure 1A shows that the two SUV curves are followed for the first approximately 10 min (with marginally lower SUV values for Hypnorm–Dormicum), but that thereafter, there is a marked increase in SUV for Hypnorm–Dormicum, but not for isoflurane. An explanation could be that although the Hypnorm–Dormicum rats lay still in the scanner, had uniform monitoring values, were negative for the tail reflex, and received supplementary injections as recommended for stable anesthesia [45], the anesthesia gradually wore off due to liver metabolism of the drugs so that they were finally lighter anesthetized than the isoflurane rats that continuously inhaled the anesthetic gas. Figure 1 thus indicates that more uniform anesthesia is achieved using isoflurane than Hypnorm–Dormicum anesthesia in the rats during the [^18^F]FDG scan. In addition to brain data, when we evaluate the [^18^F]FDG uptake in the heart, we could see that the isoflurane data corroborated previous studies with higher uptake than injected anesthesia [46]. Volatile anesthetics, such as sevoflurane and isoflurane, have been shown to protect ischemic myocardium through the activation of ATP-sensitive potassium channels (K(ATP)) and vasodilatation [47]. Moreover, several clinical trials have demonstrated their ability to prevent myocardial damage during surgical procedures, being described as responsible for cardioprotective effects [48] that explain the increase in [^18^F]FDG uptake.

Based on this study, it is not possible to unequivocally point to either isoflurane or Hypnorm–Dormicum as the best anesthetic for [^18^F]FDG PET scans of rat brains. However, the study indicates that Hypnorm–Dormicum is suitable for cortical studies, as these are not affected by the anesthesia to the same extent as with isoflurane. This is consistent with Hypnorm–Dormicum providing neuroleptanalgesia, as opposed to the cortical depression of isoflurane. Conversely, other structures (dorsomedial periaqueductal gray and molecular layer of cerebellum) in the brain, on the other hand, seem to be depressed to a greater extent by the Hypnorm–Dormicum. The timing of the scan after administration of [^18^F]FDG is less affected by isoflurane than by Hypnorm–Dormicum—because isoflurane has the advantage of being administered continuously, while injection anesthesia is given at intervals. Injecting tracers into awake animals, which are only later anesthetized and scanned, could alleviate some of the anesthesia-related problems.

### 4.5. Limitations

A major limitation of this study is that it was only performed on one sex (females). However, we are not aware of any sex-dependent anesthetic effects in similar studies investigating PET neuroimaging in experimental animals [2]. The only known study dealing with sex differences in behavioral post-anesthesia in rats anesthetized with isoflurane and propofol is thus not relevant to our study, which only deals with rats under anesthesia [49]. Another limitation is the low number of rats in each group, which, for 3R replacement reasons, was set to the lowest recommended based on Festing (2018) [28]. However, the results were so significant that a larger number of experimental animals would hardly have changed the conclusions. Finally, it is a limitation that awake rats were not included in the study—it is thus impossible to know whether either isoflurane or the Hypnorm–Dormicum anesthesia comes closest to the [^18^F]FDG uptake in awake experimental rats. However, it is likely that the uptake of [^18^F]FDG will be higher in the brains of awake rats than in rats anesthetized with the two anesthetic methods. Furthermore, in future studies, plasma glucose measurements could be included, as this could decide if the differences in [^18^F]FDG uptake were partly influenced by anesthesia effects on plasma glucose concentration. Standardized fasting should also be considered, although, for animal welfare reasons, it should be short. Future studies could advantageously include [^15^O]water scans to determine cerebral blood flow. Furthermore, could it be considered to supplement the scans with ex vivo autoradiography and immunohistochemistry, as this will provide more detailed images of the [^18^F]FDG uptake in the brain.

## 5. Conclusions

This study showed that isoflurane versus Hypnorm–Dormicum anesthesia affects the cerebral uptake of [^18^F]FDG in rats. This is in agreement with previous studies involving other anesthetics and tracers. The study emphasizes the importance of the choice of anesthesia, that it is performed uniformly, and that it is always reported in scientific publications. The study shows that a lower cortical uptake of [^18^F]FDG is achieved during the 1 h of scanning under isoflurane than under Hypnorm–Dormicum anesthesia—probably because of the effects of isoflurane on cortical activity as described before. However, [^18^F]FDG uptake may be stable at a lower level than in awake rats, and here, it is possible that Hypnorm–Dormicum better reflects the awake [^18^F]FDG uptake.

In conclusion, based on both prior research and our findings, maintaining animals awake after [^18^F]FDG administration appears preferable for preserving neuronal function, especially in cortical regions, and for approximating biological conditions more closely. However, if dynamic imaging is necessary, careful consideration of study design, including the choice of anesthesia, is essential. Selecting the appropriate [^18^F]FDG uptake condition (anesthesia vs. awake) is critical in small animal [^18^F]FDG-PET studies, as altered baseline brain glucose metabolism levels could influence the biological effects researchers aim to observe.

For future studies, it should be considered to include standardized fasting, measurements of plasma glucose before and after scanning, measurement of cerebral blood flow, and supplement the PET scans with ex vivo analysis.

## Figures and Tables

**Table 1:** Body weight, [^18^F]FDG doses, and monitoring parameters (pulse, respiration rate, and body temperature) for the twelve rats included in the study. Data are given as mean (SD).

Parameter	Isoflurane	N	Hypnorm–Dormicum	N	*p*-Value
Weight [g]	274 (20)	6	273 (23)	6	0.937
[^18^F]FDG [MBq]	29 (6)	6	28 (4)	6	0.822
Pulse [min^−1^]	381 (22)	6	371 (17)	6	0.376
Respiration rate [min^−1^]	48 (10)	6	58 (12)	6	0.125
Temperature [°C]	36.9 (0.2)	6	36.5 (0.6)	6	0.113

**Table 2:** Statistical parametric mapping outcome for the clusters.

	Cluster Level		Voxel Level		Coordinates			Brain Area
	PFWE-Corr	kE	T	Puncorr	x	y	z	
**Isoflurane < Hypnorm–Dormicum**	<0.0001	20,055	12.15	<0.0001	5.4	0.8	−4.2	Right Primary somatosensorial cortex
			9.6	<0.0001	−3.4	2.4	−1.6	Left Primary motor cortex
			5.49	<0.0001	3.4	−6.6	−1	Right Primary motor cortex
	<0.0001	1207	8.25	<0.0001	−3	−6	−1	Left visual cortex
	0.025	465	6.08	<0.0001	7	−3	−3.6	Right Primary motosensorial cortex
**Isoflurane > Hypnorm–Dormicum**	<0.0001	6480	7.44	<0.0001	0.2	−5.6	−5.8	Dorsomedial periaqueductal gray
	0.088	313	5.86	<0.0001	−0.4	−11.8	−2	Left molecular layer cerebellum

## Data Availability

Data can be accessed by contacting the authors.
